# A Case Report of a Bilateral Carotid Body Tumor and a Review of Its Imaging Manifestations

**DOI:** 10.5334/jbsr.2952

**Published:** 2023-04-06

**Authors:** Long Xu, Yao Kang, Xing Wen

**Affiliations:** 1 Ziyang People’s Hospital, CN

**Keywords:** carotid body tumor, bilateral, CT, imaging manifestations

## Abstract

Bilateral carotid body tumors (CBTs) are extremely uncommon. In recent years, some clinical cases have been documented. A case of bilateral CBT is reported with a review of its imaging manifestations. When a unilateral CBT is discovered, assessment of the contralateral site is essential. The ‘goblet’ sign is a characteristic imaging feature of CBT. The preferred imaging modality is computed tomography angiography.

**Teaching Point:**

When a carotid body tumor is suspected on one side, careful examinationof the contralateral side is essential; The preferred imaging approach is CTA.

## Introduction

Carotid body tumors (CBTs) are rare paragangliomas that occur at the bifurcation of the carotid artery [1]. Clinical diagnosis is usually confirmed by imaging. In recent years, some clinical cases have been documented, yet without detailed review of the imaging features.

## Case History

**Figure 1:**
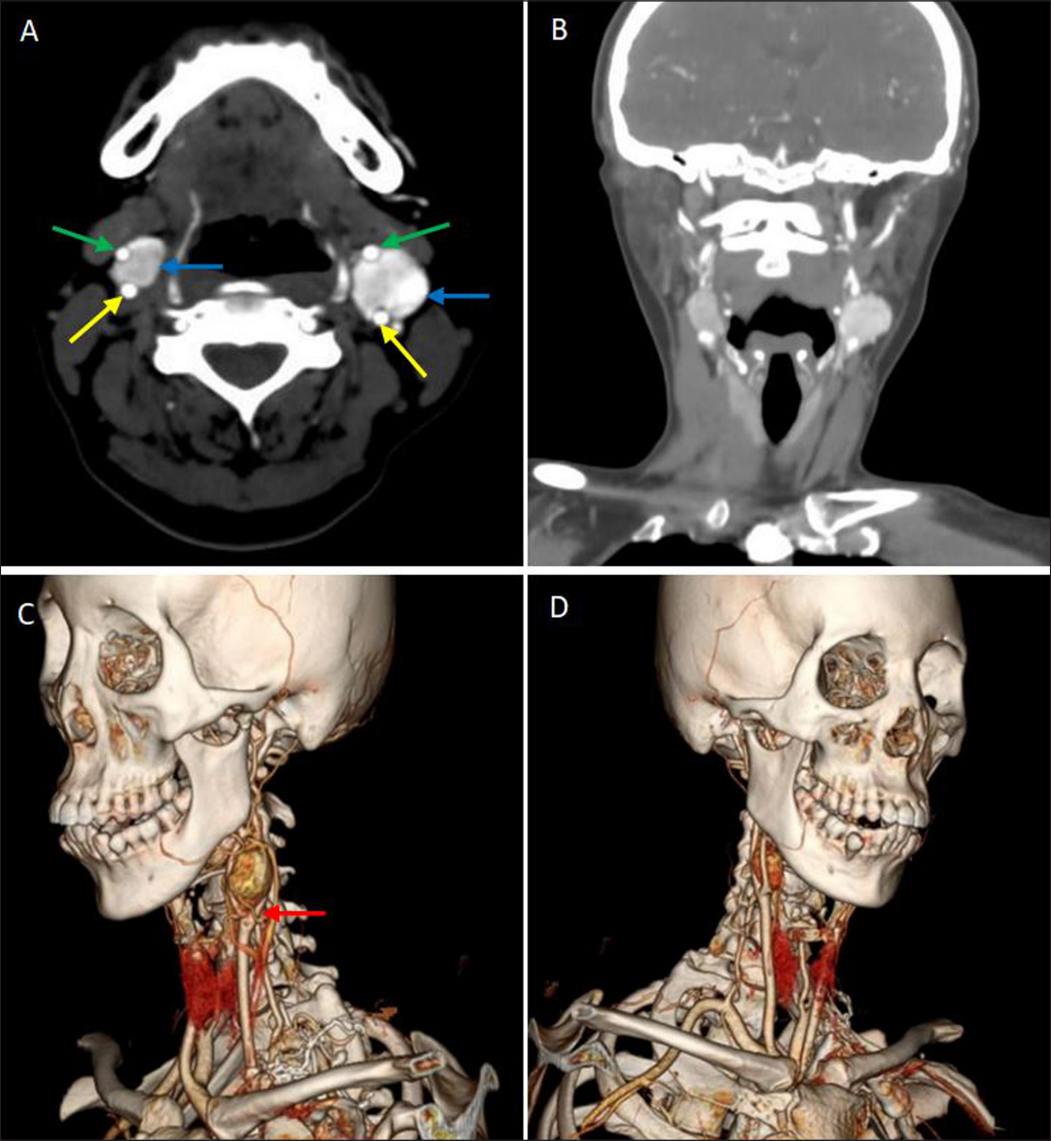


A 50-year-old female incidentally palpated a well-circumscribed painless mass on the left side of the neck. She had no relevant medical history. There was no follow-up for two years. The patient then consulted because of steady growth of the mass. A CT scan revealed unexpectedly bilateral masses at carotid bifurcation. The computed tomography angiography (CTA) shows the bilateral masses (blue arrows in Figure 1). The internal carotid artery (yellow arrows) and external carotid artery (green arrows) were compressed by the CBTs. In volume rendering (VR) images, the ‘goblet’ sign (Figure 1, red arrow) was clearly visible.

**Figure 2:**
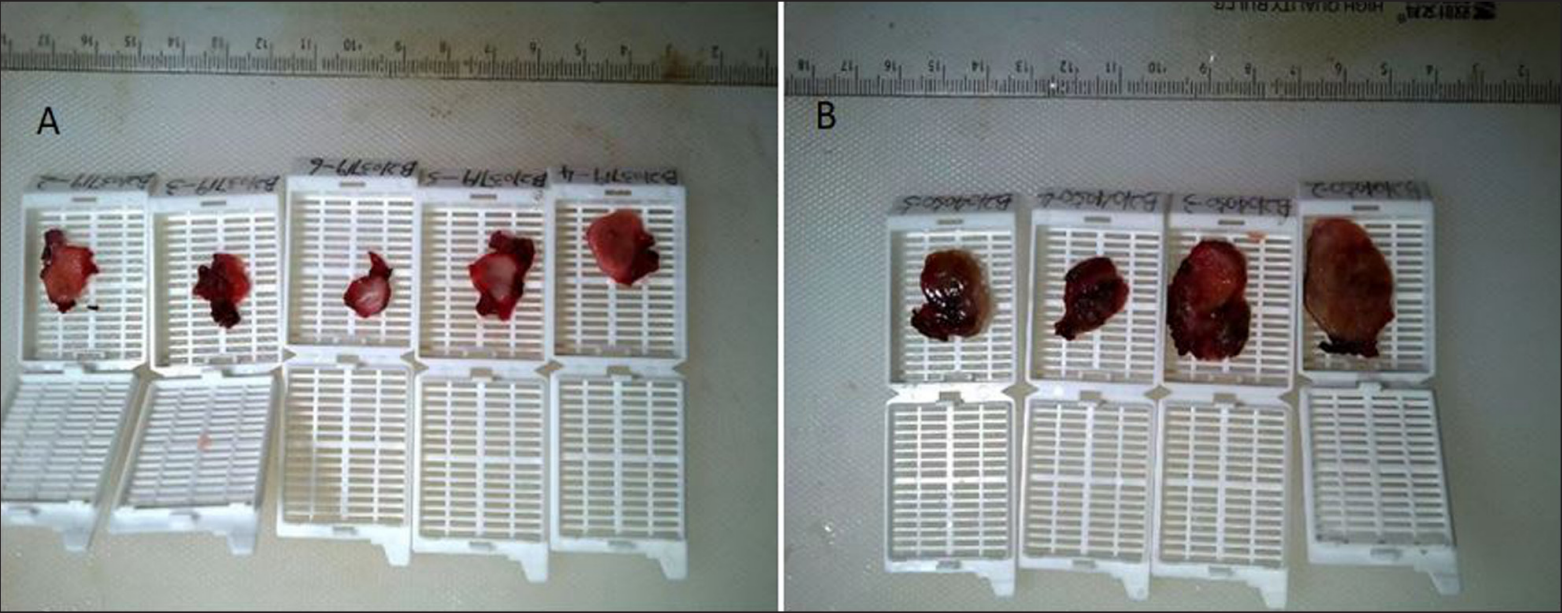


**Figure 3:**
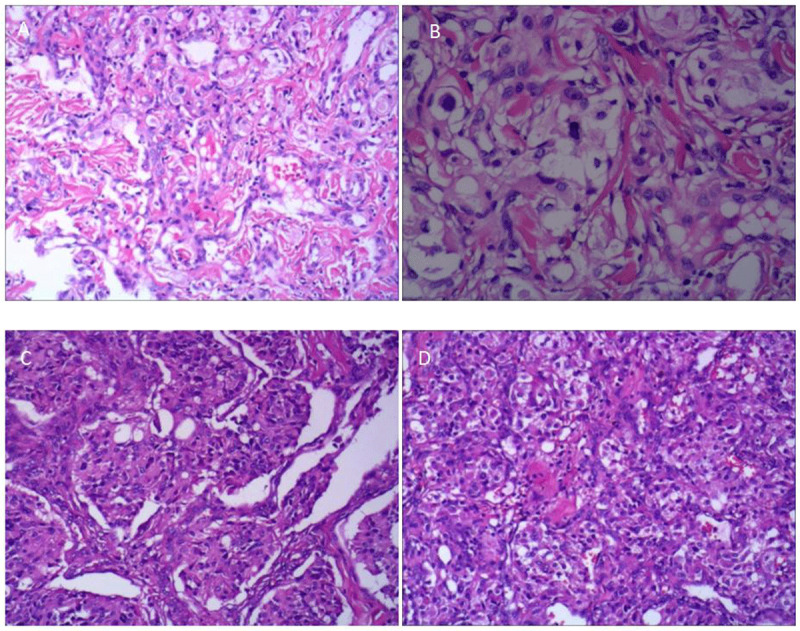


The mass on the right side was surgically removed and the contralateral mass was removed three weeks later. Both lesions were pathologically proven CBTs. The bilateral CBTs have a brownish appearance (Figure 2, A left, B right CBT). Histologically, show the classic architectural pattern of paragangliomas, consisting of well-defined solid nests of tumor cells (Figure 3 A and C; H&E stain; X40). The cytological characteristics of the tumor cells include with somewhat pleomorphic nuclei and extensive granular cytoplasm (Figure 3 B and D; H&E stain; X200).

Color-Doppler ultrasonography is non-invasive and may clarify the size of a mass and the relationship between the mass and surrounding blood vessels, as well as distinguishing CBTs from lymphadenopathy or other space-occupying neck lesions [2]. However, color-Doppler ultrasonography doesn’t allow accurate Shamblin classification and is only used for initial screening.

Digital subtraction angiography (DSA) is the gold standard for the diagnosis of CBTs, which are characterized by reticular or mass shadows at the bifurcation of the carotid artery, the compression of the adjacent internal and external carotid artery, and the widening of the bifurcation of the carotid artery, that is, the ‘goblet’ sign. DSA can clearly identify the origin of the feeding artery [3].

The frequent CT manifestation of CBTs is a soft tissue attenuating mass at the carotid bifurcation, with distinct boundaries, mostly with relatively uniform density, and a low-density necrotic area in larger masses. Most masses are obviously enhancing in the arterial phase. Three-dimensional reconstructions clearly outline the ‘goblet’ sign. Multiplanar reconstruction (MPR) and VR techniques clearly show the location of CBT and its relationship with adjacent structures, as well as preliminar Shamblin typing [4,5,6]. Based on enhanced images, the degree of vascularity and the presence of internal necrosis can be observed, enabling assessment of the tumor composition. In addition, VR reconstruction can identify the supplying artery and observe the anatomy from multiple angles, contributing to the therapeutic management [5].

There are few reports on MRI in the diagnosis of CBTs. MRI delineates the extent of a tumor, its vascularity, the condition of the carotid vessels, and the presence of other swellings or enlarged nodes and is a good alternative to CT [7].

## Conclusion

When a CBT is suspected on one side, careful examination of the contralateral side is essential. The preferred imaging approach is CTA. The feeding artery can be identified using DSA. MRA may be used for vascular mapping. Color-Doppler ultrasonography is mainly useful for initial screening.
